# Cancer risk in Swedish women: the relation to size at birth

**DOI:** 10.1054/bjoc.2000.1738

**Published:** 2001-05

**Authors:** S W Andersson, C Bengtsson, L Hallberg, L Lapidus, A Niklasson, A Wallgren, L Hulthén

**Affiliations:** Department of Clinical Nutrition, Göteborg University, SE 413 45 Göteborg, Sweden; Department of Primary Health Care, Göteborg University, SE 411 33 Göteborg, Sweden; Department of Medicine, Sahlgrenska University Hospital, SE 413 45 Göteborg, Sweden; Göteborg Pediatric Growth Research Centre, Institute for the Health of Women and Children, Göteborg University, SE 413 45 Gteborg, Sweden; Department of Oncology, Göteborg University, SE 413 45 Göteborg, Sweden

**Keywords:** adult, birth weight, cancer, epidemiology, prenatal, women

## Abstract

The relationship between fetal growth as indicated by weight and length at birth, and cancer risk in 1080 adult Swedish women was examined. Birth factors were retrieved from original midwife records for the years 1914, 1918, 1922 and 1930, and primary cancer cases were identified by matching with national and regional cancer registries through the year 1998. A positive and statistically significant increased risk for cancer was found with increasing birth weight or birth length for all site cancer and non-hormone related cancer, defined as all cancer sites excluding breast, uterus and ovary. Addition of factors suspected to influence cancer risk, maternal proteinuria, birth order, own parity and age at menarche, did not attenuate this relation. Previously only breast cancer has been reported to be related to size at birth in adult women and this is the first study to report that cancer sites other than the major hormone-related sites may be influenced by size at birth, as measured by either weight or length at birth; these findings warrant further investigation. © 2001 Cancer Research Campaign http://www.bjcancer.com


*British Journal of Cancer* (2001) **84**(9), 1193–1198
© 2001 Cancer Research Campaign
doi: 10.1054/ bjoc.2001.1738, available online at http://www.idealibrary.com on IDEAL

http://www.bjcancer.com

### Cancer risk in Swedish women: the relation to size at birth

**SW Andersson[1], C Bengtsson[2], L Hallberg[1], L Lapidus[3], A Niklasson[4], A Wallgren[5] and L Hulthén[1]**

[1]Department of Clinical Nutrition, Göteborg University, SE 413 45 Göteborg, Sweden; [2]Department of Primary Health Care, Göteborg University, SE 411 33 Göteborg, Sweden; [3]Department of Medicine, Sahlgrenska University Hospital, SE 413 45 Göteborg, Sweden; [4]Göteborg Pediatric Growth Research Centre, Institute for the Health of Women and Children, Göteborg University, SE 413 45 Göteborg, Sweden; [5]Department of Oncology, Göteborg University, SE 413 45 Göteborg, Sweden

**Summary** The relationship between fetal growth as indicated by weight and length at birth, and cancer risk in 1080 adult Swedish women was examined. Birth factors were retrieved from original midwife records for the years 1914, 1918, 1922 and 1930, and primary cancer cases were identified by matching with national and regional cancer registries through the year 1998. A positive and statistically significant increased risk for cancer was found with increasing birth weight or birth length for all site cancer and non-hormone related cancer, defined as all cancer sites excluding breast, uterus and ovary. Addition of factors suspected to influence cancer risk, maternal proteinuria, birth order, own parity and age at menarche, did not attenuate this relation. Previously only breast cancer has been reported to be related to size at birth in adult women and this is the first study to report that cancer sites other than the major hormone-related sites may be influenced by size at birth, as measured by either weight or length at birth; these findings warrant further investigation. © 2001 Cancer Research Campaign http://www.bjcancer.com

**Keywords**: adult; birth weight; cancer; epidemiology; prenatal; women

Known risk factors for various cancer forms can only partly explain the incidence of cancer in adult populations. There is general agreement that non-genetic factors occurring in adult life, including exposure to smoke or pollutants, dietary factors, occupational exposure and viral infections, are the predominant contributors to cancer causation. Only about 5–10% of all cancer can be attributed to dominant genes or inherited cancer syndromes (Lynch et al, 1997). The prenatal or in utero environment has only recently been examined for its role in cancer in adults (Ekbom, 1998).

During the past decade, it has been observed that several chronic diseases in adulthood may be a consequence of influences occurring during the period of gestational development (Joseph and Kramer, 1996). Size at birth, an indicator of conditions during fetal development, may be an important factor in cancer pathogenesis. Previous studies relating size at birth to adult cancer have focused on, what may be termed, hormone-related cancers, that is, breast, uterine, ovarian, prostate and testicular cancer (Brown et al, 1986; Ekbom et al, 1992, 1996, 1997; Michels et al, 1996; Sanderson et al, 1996; Tibblin et al, 1995). The relation between size at birth and other adult cancers has not been reported in the literature, while among children, high birth weight has been associated with certain cancers including neuroblastoma, Wilms' tumour, leukaemia and brain tumours (Daling et al, 1984; Yeazel et al, 1997).

The relation between size at birth, as indicated by birth weight and birth length, and cancer risk was assessed in a population study of adult Swedish women.

*Received 25 September 2000*
*Revised 23 January 2001*
*Accepted 25 January 2001*

*Correspondence to:* SW Andersson

### METHODS

The present investigation is based on a prospective study initiated in 1968 of 4 birth cohorts of altogether 1260 women born on selected dates in the years 1914, 1918, 1922 and 1930 residing in Göteborg at the study start (Bengtsson et al, 1973). We complemented the original population study material by tracing all females born in Göteborg on the same dates who had survived to at least 15 years of age but who had moved out of the area before the study onset in 1968 and/or had died before 1968 (*n* 479). The women included in the study were of singleton birth, born in Sweden (*n* 1739).

Original midwife records for both home and hospital deliveries were traced in city and regional archives in Sweden. Measures of birth weight, birth length and other birth factors were available dependent on year of birth and delivery site (Andersson et al, 2000b). Demographic variables and maternal characteristics were derived from midwife records (gestational age, maternal proteinuria, maternal age, birth order), parish records (maternal age, birth order, own parity), and by questionnaire prior to health examinations in 1968 and 1974 (prospective population study) or sent home in 1995 (complementary population) to attain information on age at menarche and own parity. The questionnaire was reviewed at each health examination with a study nurse or by telephone interview (complementary population) to assure completeness of data collection. Missing data on age at menarche is mainly for the complementary population who were deceased before 1995.

At the time of the study, the Swedish National Cancer Register was complete up to 1997 and the Regional Cancer Register for western Sweden through 1998. Since 1958, the attending physician must report all newly diagnosed cases of cancer to one of 6 regional cancer registries covering the whole country, and a separate report is required from the pathologist or cytologist (Socialstyrelsen, 1997).

These reports are compiled at both the regional and national level. By means of the unique 10-digit personal number assigned to all residents of Sweden, the population study database could be matched with the cancer registries to identify primary incident cases. Date of death (from any cause) was determined by matching personal identification numbers with the Swedish Cause of Death Registry and by confirmation with parish records.

Cancer cases were analysed as combined all site cancer morbidity and also divided into 'hormone-related' and 'non-hormonal' cancers and are referred to as such in the following analysis and discussion; the former comprises cancers of the breast, uterus and ovaries, the major cancer sites with a hormonal aetiology (Miller, 1978). Non-hormonal cancers, as defined here, are thus all other cancer sites. As there is evidence that cancer of the colon may be hormone-related (Potter, 1995), the defined hormone-related sites and colon cancer are also examined in combination. Cancers of the 'digestive system', ICD7 codes 150–158, are analysed separately to examine a sub-group of the 'non-hormone-related' cancers.

This study was approved by the Research Ethics Committee of Göteborg University.

### Statistical methods

Characteristics of participants and non-participants were compared using a 2-sample *t*-test for continuous variables and chi-squared test for proportions for factors which may influence cancer risk or birth outcome – maternal age, birth order of the participant, own parity (defined as number of pregnancies) and age of menarche of the participants. Birth weight and birth length were analysed as continuous variables and in population quintiles birth weight and tertiles birth length. Cox proportional hazards modelling was used to examine trend in cancer risk in relation to size at birth, treating weight and length separately, and in modelling cancer risk with consideration of covariates implicated in cancer pathogenesis: maternal proteinuria, birth order, own parity and age at menarche. All analyses were adjusted for gestational age as well as cohort membership to adjust for cohort effects. All individuals were included in the Cox models up to time of first cancer diagnosis, death from any cause or the cessation of the study. The validity of the proportional hazards assumption was tested and no significant effects for time dependence were found. All analyses were carried out using the SAS software release 6.12 statistical package 1989–1996 and specifically the PHREG procedure.

### RESULTS

Of the 1739 women eligible for inclusion in the study, midwife records (home and hospital deliveries) for the births of 1184 (68%) of the women were identified in which birth weight was recorded. Of these records, 1105 contained useable information on gestational age. Another 25 women were removed from further analysis as they were not alive in January 1958 when the National Cancer Register was initiated. A total of 1080 women were thus included in the present study. This represents participation by 53.4% (born 1914), 50.4% (1918), 64.2% (1922), and 77.8% (1930) of the original birth cohorts. In total 76.7% (827/1080) of the participants were born in Göteborg.

There was no statistically significant difference in age at menarche or own parity in women without known birth weight or gestational age from midwife records and those included in the study. Women included in the study had slightly younger mothers ($P < 0.05$) (Table 1a) and were subsequently of lower birth order ($P < 0.001$) (Table 1b). Ages ranged from 28 (1930 cohort, age in 1958) to 84 years (1914 cohort, age in 1998). Mean, standard deviation and range of birth weight per quintile and birth length per tertile are shown in Table 2.

In total, 262 primary cancer cases (24.3% of the 1080 women) were identified from the matching of our database with that of the Swedish National Cancer Registry (1958–1997) and the Regional Cancer Registry (1958–1998) (Table 3). The distribution of cancer cases per birth weight quintile and birth length tertile is presented in Table 2.

### Birth weight and cancer risk

### *Univariate analysis*

A significant and positive trend was found between birth weight, as a continuous variable, and all cancers ($P = 0.006$) and non-hormonal cancers ($P = 0.003$). A positive trend was also found between birth weight and combined hormone-related cancers, breast cancer alone, cancers of the digestive system and for hormone-related cancer together with colon cancer, but these relations were not statistically significant.

### *Modelling cancer risk and birth weight*

Cancer risk (all sites combined) was significantly higher in the 3 highest birth weight quintiles in relation to the lowest quintile of birth weight, an increase in risk by 54–71% adjusted for gestational age (Table 4). When separately analysing combined hormone-related cancers or breast cancer alone and accounting for gestational age, a positive but statistically non-significant association was observed between birth weight and cancer risk. However, when only the non-hormonal cancers were included in the analysis, the statistically significant positive relation to birth weight remained. A 2-fold increase in cancer risk was found in the highest quintile of birth weight with reference to the lowest quintile (Table 4), (RR 2.07, 95% CL 1.22, 3.50, adjusted for gestational age). The subgroup of digestive cancer found a more than 2-fold risk for cancer in the highest birth weight quintile compared to the lowest, however not statistically significant. A similar trend was found for hormone-related cancer sites together with colon cancer.

Addition of covariates to the models did not have a marked effect on the relation between birth weight and cancer risk (Table 4). Although not statistically significant, slightly different effects are seen dependent on whether hormonal or non-hormonal cancers are included in the analysis. For hormone-related cancers, birth order and age at menarche had the most influence on this relation, while maternal proteinuria, birth order and own parity had the most effect on non-hormonal cancer risk.

### Birth length and cancer risk

### *Univariate analysis*

A positive and statistically significant trend was found between birth length and all cancers combined ($P = 0.007$), and non-hormonal cancers ($P = 0.011$), while a marginally significant trend was found for combined hormone-related cancers and colon cancer ($P = 0.058$), adjusted for gestational age.

### *Modelling cancer risk and birth length*

The incidence of all site cancer was statistically significantly higher in the highest tertile of birth length in reference to the

© 2001 Cancer Research Campaign

**Table 1a** Study population characteristics

| | Participants[a] | | | Women excluded[b] | | |
|---|---|---|---|---|---|---|
| | No. | Mean (SD) | Range | No. | Mean (SD) | Range |
| Demographic characteristics | | | | | | |
| Maternal age (y)[c] | 1080 | 29.2 (6.3) | 16.3–48.0 | 650 | 30.3 (6.5) | 16.5–47.7 |
| Age at menarche (y) | 912 | 13.8 (1.4) | 10–20 | 579 | 13.8 (1.3) | 10–20 |

[a]Women with original midwife records and known gestational age. [b]Women without traced midwife records or with missing gestational age. [c]*t*-test for heterogeneity, $P < 0.05$.

**Table 1b** Study population characteristics

| | Participants[a] | | Women excluded[b] | |
|---|---|---|---|---|
| | No. | % | No. | % |
| Demographic characteristics | | | | |
| Birth order[c d] | | | | |
| 1 | 419 | 38.8 | 157 | 28.8 |
| 2–3 | 406 | 37.5 | 193 | 35.4 |
| ≥4 | 255 | 23.7 | 196 | 35.8 |
| Parity[e] | | | | |
| 0 | 177 | 16.4 | 132 | 20.2 |
| 1–2 | 542 | 50.3 | 314 | 48.1 |
| ≥3 | 357 | 33.2 | 207 | 31.7 |

[a]Women with original midwife records and known gestational age. [b]Women without traced midwife records or with missing gestational age. [c]Missing for 113 women excluded from study. [d]chi-squared test for heterogeneity, $P < 0.001$. [e]Missing for 4 participants and 6 excluded women.

**Table 2** Birth weight, birth length and cancer cases (no.) by cancer site per birth weight quintile (Q) and birth length tertile (T)

| | Birth weight (g) | | | | Cancer site (no. of cases) | | | | | |
|---|---|---|---|---|---|---|---|---|---|---|
| | No. | Mean | SD | Range | All site | Hormonal | Breast | Non-hormonal | Digestive | Hormonal + colon |
| Q1 | 217 | 2745 | 277 | 1600–3000 | 39 | 17 | 9 | 22 | 6 | 21 |
| Q2 | 207 | 3204 | 92 | 3010–3349 | 45 | 18 | 10 | 27 | 9 | 23 |
| Q3 | 206 | 3475 | 62 | 3350–3590 | 54 | 25 | 14 | 29 | 5 | 27 |
| Q4 | 217 | 3738 | 99 | 3600–3960 | 57 | 21 | 14 | 36 | 9 | 24 |
| Q5 | 233 | 4241 | 285 | 4000–5500 | 67 | 23 | 15 | 44 | 14 | 32 |
| All | 1080 | 3495 | 547 | 1600–5500 | 262 | 104 | 62 | 158 | 43 | 127 |
| | Birth length (cm) | | | | | | | | | |
| T1 | 292 | 47.6 | 2.1 | 35–49 | 55 | 22 | 11 | 33 | 11 | 27 |
| T2 | 226 | 50.0 | 0.2 | 49.5–50.5 | 44 | 17 | 12 | 27 | 6 | 19 |
| T3 | 354 | 52.4 | 1.7 | 51–60 | 99 | 35 | 20 | 64 | 19 | 47 |
| All | 872 | 50.2 | 2.6 | 35–60 | 198 | 272 | 43 | 124 | 36 | 93 |

SD, standard deviation.

lowest tertile (Table 5), an increase of about 60% adjusted for gestational age. For hormone-related cancer sites combined and breast cancer alone, respectively, cancer risk increased by 35% and 48% in the highest tertile birth length in reference to the lowest, however not statistically significant. Analysis of non-hormonal cancer revealed a significant positive relation between birth length and cancer risk. This reflects a 72% increase in risk in the highest tertile in relation to the lowest tertile birth length (RR 1.72, 95% CL 1.12, 2.66 adjusted for gestational age). There was no significant increase in risk for digestive cancer across birth length tertiles. The combination of hormone-related cancer and colon cancer resulted in a raised cancer risk in tertile III compared to tertile I, however not statistically significant (RR 1.57, 95% CL 0.96, 2.56). The addition of covariates in the Cox models did not have any marked effect on the relations with all cancer sites combined (Table 5). However, analysis of the subdivisions of cancer sites revealed a different picture with respect to the covariate age at menarche. Inclusion of age at menarche in the models resulted in a 62% increase in hormone-related cancer risk in the highest birth length tertile compared to the lowest tertile,

*© 2001 Cancer Research Campaign*

**Table 3** Frequency of cancer diagnosis in 1080 Swedish women

| Cancer site | ICD-7 | No. of cases |
|---|---|---|
| Hormone related | | |
| Breast | 170 | 62 |
| Female reproductive organs | 172–176 | 42 |
| Non-hormone related | | |
| Ear, nose & throat | 140–148, 160, 161 | 5 |
| Digestive system (total) | 150–158 | 43 |
| Colon (alone) | (153, 154) | (23) |
| Lung | 162, 163 | 19 |
| Urinary system | 180–181 | 9 |
| Malignant melanoma | 190 | 6 |
| Nervous system | 193 | 14 |
| Endocrine system | 194, 195 | 10 |
| Leukaemia & lymphoma | 200–209 | 23 |
| Others | 164, 171, 191–192,196–199 | 29 |
| | Total cases | 262 |

although not statistically significant (RR 1.62 95% CL 0.86, 3.06, *P* for trend = 0.12). For breast cancer risk there was a 2-fold increase in risk in the highest length tertile compared to the lowest (RR 2.21 95% CL 0.88, 5.53, *P* for trend = 0.09). With hormonal and colon cancers combined, inclusion of age at menarche resulted in a significantly increased cancer risk in the highest birth length tertile (RR 1.80 95% CL 1.02, 3.17). Inclusion of age at menarche in non-hormonal and digestive cancer models resulted in a slight decrease in cancer risk for these cancer forms.

### DISCUSSION

High weight or length at birth was associated with a significant increase in risk for cancer in adulthood. To look at more specific divisions of cancer sites, analyses were carried out at the level of non-hormonal and hormonal-associated cancer sites. Non-hormonal cancer sites combined (all sites other than the breast, uterus or ovary) showed a linear and statistically significant increase in cancer risk from the lowest to highest birth weight quintile and birth length tertile, a relation not previously reported. The different association patterns for non-hormonal and hormonal-associated cancers suggest that several factors may be involved in the relationship, working in different directions, of possibly varying importance and mechanistic expression dependent on cancer site.

In 1990 a role for size at birth in breast cancer morbidity was hypothesized (Trichopoulos, 1990). Indications were reported of a positive (though not significant) relationship between weight at birth and breast cancer (Ekbom et al, 1992, 1997) as was also found in a recent British cohort (Stavola et al, 2000) and in the current study. An increased risk for prostate cancer has been found with high birth weights (Ekbom et al, 1996; Tibblin et al, 1995),

**Table 4** Cancer risk by quintile[a] birth weight, singleton births, with known gestational age (*n* = 1080 women)

| | Rate ratio (95% confidence limits) | | | | |
|---|---|---|---|---|---|
| | Q2 | Q3 | Q4 | Q5 | *P* for trend |
| **All sites**[b] (Cases = 262) | 1.27 (0.82, 1.96) | 1.54 (1.01, 2.33) | 1.61 (1.06, 2.44) | 1.71 (1.14, 2.56) | 0.0054 |
| [c] + maternal proteinuria | 1.38 (0.88, 2.16) | 1.57 (1.02, 2.42) | 1.68 (1.09, 2.60) | 1.77 (1.16, 2.70) | 0.0064 |
| +birth order | 1.26 (0.82, 1.94) | 1.50 (0.98, 2.28) | 1.56 (1.02, 2.37) | 1.60 (1.05, 2.43) | 0.0189 |
| +own parity | 1.29 (0.83, 1.99) | 1.57 (1.03, 2.39) | 1.63 (1.07, 2.49) | 1.74 (1.16, 2.62) | 0.0046 |
| +age at menarche[d] | 1.39 (0.86, 2.23) | 1.37 (0.85, 2.22) | 1.53 (0.95, 2.45) | 1.66 (1.05, 2.63) | 0.0343 |
| **Hormonal**[b,e] (Cases = 104) | 1.12 (0.56, 2.19) | 1.56 (0.84, 2.92) | 1.29 (0.67, 2.49) | 1.28 (0.67, 2.42) | 0.427 |
| [c] + maternal proteinuria | 1.13 (0.57, 2.25) | 1.51 (0.79, 2.88) | 1.28 (0.65, 2.51) | 1.30 (0.67, 2.51) | 0.431 |
| +birth order | 1.12 (0.57, 2.19) | 1.55 (0.83, 2.91) | 1.28 (0.66, 2.48) | 1.25 (0.65, 2.42) | 0.479 |
| +own parity | 1.09 (0.56, 2.13) | 1.54 (0.82, 2.87) | 1.25 (0.65, 2.41) | 1.27 (0.67, 2.41) | 0.430 |
| +age at menarche[d] | 1.06 (0.50, 2.22) | 1.17 (0.56, 2.43) | 1.21 (0.58, 2.49) | 1.38 (0.69, 2.76) | 0.325 |
| **Breast**[b] (Cases = 62) | 1.16 (0.47, 2.87) | 1.65 (0.71, 3.86) | 1.58 (0.67, 3.72) | 1.57 (0.67, 3.64) | 0.228 |
| [c]+maternal proteinuria | 1.18 (0.45, 3.07) | 1.69 (0.69, 4.12) | 1.73 (0.71, 4.22) | 1.63 (0.67, 3.97) | 0.198 |
| +birth order | 1.14 (0.46, 2.84) | 1.59 (0.68, 3.73) | 1.51 (0.64, 3.58) | 1.43 (0.60, 3.41) | 0.348 |
| +own parity | 1.14 (0.46, 2.83) | 1.64 (0.70, 3.82) | 1.56 (0.66, 3.68) | 1.57 (0.68, 3.66) | 0.223 |
| +age at menarche[d] | 1.13 (0.39, 3.29) | 1.53 (0.56, 4.21) | 1.76 (0.66, 4.69) | 1.93 (0.75, 5.00) | 0.105 |
| **Non-hormonal**[b,f] (Cases = 158) | 1.39 (0.78, 2.46) | 1.50 (0.85, 2.63) | 1.87 (1.08, 3.22) | 2.07 (1.22, 3.50) | 0.0033 |
| [c]+maternal proteinuria | 1.59 (0.89, 2.87) | 1.61 (0.90, 2.90) | 2.03 (1.15, 3.59) | 2.19 (1.26, 3.80) | 0.0042 |
| +birth order | 1.37 (0.77, 2.43) | 1.44 (0.82, 2.54) | 1.78 (1.03, 3.08) | 1.88 (1.09, 3.23) | 0.0143 |
| +own parity | 1.45 (0.81, 2.59) | 1.58 (0.89, 2.79) | 1.95 (1.12, 3.40) | 2.14 (1.25, 3.66) | 0.0028 |
| +age at menarche[d] | 1.68 (0.90, 3.14) | 1.53 (0.80, 2.91) | 1.81 (0.97, 3.38) | 1.92 (1.04, 3.55) | 0.0042 |
| **Digestive system**[b] (Cases = 43) | 1.73 (0.60, 4.94) | 0.97 (0.29, 3.23) | 1.74 (0.60, 5.04) | 2.47 (0.92, 6.62) | 0.0829 |
| [c] + maternal proteinuria | 2.07 (0.68, 6.29) | 1.04 (0.30, 3.66) | 1.82 (0.59, 5.62) | 2.22 (0.76, 6.50) | 0.2214 |
| + birth order | 1.68 (0.59, 4.81) | 0.89 (0.27, 2.97) | 1.56 (0.53, 4.54) | 1.97 (0.71, 5.47) | 0.2379 |
| + own parity | 1.70 (0.59, 4.86) | 0.96 (0.29, 3.20) | 1.71 (0.59, 4.96) | 2.48 (0.93, 6.66) | 0.0792 |
| + age at menarche[d] | 1.57 (0.53, 4.67) | 0.63 (0.15, 2.56) | 1.19 (0.37, 3.84) | 1.52 (0.51, 4.58) | 0.6558 |
| **Hormonal + colon**[b] (Cases = 127) | 1.20 (0.66, 2.19) | 1.42 (0.79, 2.53) | 1.25 (0.69, 2.28) | 1.51 (0.86, 2.67) | 0.182 |
| [c] + maternal proteinuria | 1.26 (0.68, 2.35) | 1.40 (0.76, 2.56) | 1.25 (0.67, 2.34) | 1.51 (0.84, 2.73) | 0.227 |
| + birth order | 1.20 (0.66, 2.19) | 1.42 (0.79, 2.53) | 1.25 (0.68, 2.28) | 1.50 (0.84, 2.68) | 0.208 |
| + own parity | 1.17 (0.64, 2.14) | 1.41 (0.79, 2.51) | 1.22 (0.67, 2.23) | 1.51 (0.86, 2.66) | 0.177 |
| + age at menarche[d] | 1.11 (0.57, 2.14) | 1.03 (0.52, 2.02) | 1.18 (0.61, 2.27) | 1.37 (0.74, 2.57) | 0.318 |

[a] Where quintile 1 (Q1) is reference. [b]Adjusted for cohort membership and gestational age. [c]Further adjusted for listed factor. [d]Age at menarche available for only 912 women. [e]Where hormonal = breast, uterine and ovarian cancers and [f]non-hormonal = all other sites.

 *© 2001 Cancer Research Campaign*

**Table 5** Cancer risk by tertile[a] birth length (*n* = 872 women)

| | Rate ratio (95% confidence limits) | | |
|---|---|---|---|
| | **T2** | **T3** | ***P* for trend** |
| **All sites**[b] (cases = 198) | 1.13 (0.76, 1.69) | 1.57 (1.12, 2.21) | 0.0071 |
| [c]+ maternal proteinuria | 1.16 (0.77, 1.75) | 1.57 (1.11, 2.24) | 0.0090 |
| + birth order | 1.13 (0.75, 1.69) | 1.54 (1.09, 2.18) | 0.0110 |
| + own parity | 1.14 (0.76, 1.70) | 1.56 (1.11, 2.19) | 0.0082 |
| + age at menarche[d] | 1.00 (0.63, 1.58) | 1.54 (1.06, 2.25) | 0.0178 |
| **Hormonal**[b,e] (cases = 74) | 1.06 (0.56, 2.02) | 1.35 (0.78, 2.33) | 0.272 |
| [c]+ maternal proteinuria | 1.02 (0.53, 1.97) | 1.33 (0.76, 2.34) | 0.293 |
| + birth order | 1.06 (0.56, 2.02) | 1.33 (0.77, 2.32) | 0.295 |
| + own parity | 1.07 (0.56, 2.04) | 1.35 (0.78, 2.34) | 0.271 |
| + age at menarche[d] | 1.07 (0.50, 2.30) | 1.62 (0.86, 3.06) | 0.119 |
| **Breast**[b] (cases = 43) | 1.41 (0.61, 3.25) | 1.48 (0.70, 3.16) | 0.330 |
| [c]+ maternal proteinuria | 1.36 (0.57, 3.27) | 1.48 (0.67, 3.26) | 0.343 |
| + birth order | 1.41 (0.61, 3.25) | 1.43 (0.66, 3.06) | 0.392 |
| + own parity | 1.41 (0.61, 3.26) | 1.48 (0.70, 3.16) | 0.327 |
| + age at menarche[d] | 1.58 (0.56, 4.49) | 2.21 (0.88, 5.53) | 0.087 |
| **Non-hormonal**[b,f] (cases = 124) | 1.17 (0.70, 1.97) | 1.72 (1.12, 2.66) | 0.0107 |
| [c]+ maternal proteinuria | 1.26 (0.75, 2.14) | 1.75 (1.11, 2.74) | 0.0129 |
| + birth order | 1.17 (0.70, 1.97) | 1.68 (1.09, 2.61) | 0.0163 |
| + own parity | 1.17 (0.70, 1.97) | 1.70 (1.10, 2.63) | 0.0130 |
| + age at menarche[d] | 0.96 (0.54, 1.71) | 1.50 (0.94, 2.41) | 0.0712 |
| **Digestive system**[b] (cases = 36) | 0.83 (0.30, 2.30) | 1.65 (0.76, 3.58) | 0.169 |
| [c]+ maternal proteinuria | 0.88 (0.30, 2.30) | 1.56 (0.76, 3.58) | 0.178 |
| + birth order | 0.83 (0.30, 2.27) | 1.49 (0.68, 3.27) | 0.279 |
| + own parity | 0.85 (0.31, 2.32) | 1.66 (0.77, 3.60) | 0.165 |
| + age at menarche[d] | 0.45 (0.12, 1.65) | 1.43 (0.62, 3.30) | 0.335 |
| **Hormonal + colon**[b] (cases = 93) | 1.02 (0.56, 1.85) | 1.57 (0.96, 2.56) | 0.0581 |
| [c]+ maternal proteinuria | 1.00 (0.54, 1.86) | 1.54 (0.93, 2.56) | 0.0743 |
| +birth order | 1.02 (0.56, 1.85) | 1.55 (0.94, 2.54) | 0.0687 |
| + own parity | 1.03 (0.57, 1.86) | 1.57 (0.96, 2.57) | 0.0568 |
| + age at menarche[d] | 0.97 (0.48, 1.98) | 1.80 (1.02, 3.17) | 0.0296 |

[a] Where tertile 1 (T1) is reference [b]Adjusted for cohort membership and gestational age. [c]Further adjusted for listed factor. [d]Age at menarche available for only 752 women. [e]Where hormonal = breast, uterine and ovarian cancers and [f]non-hormonal = all other sites.

while low birth weight has been associated with testicular cancer in young adults (Brown et al, 1986).

Birth order, pregnancy, toxaemia, age at menarche and pregnancy history may be risk factors for cancer (Janerich et al, 1989; Hsieh et al, 1990; Potischman and Troisi, 1999). We therefore examined the effect of including these factors in the statistical modelling but found no marked effect on the relation between weight or length at birth and cancer in adulthood.

In Swedish studies of hospital-born infants, pre-eclampsia was found to have a ‘protective’ effect on breast cancer risk (Ekbom et al, 1992, 1997). In the present study, no marked effect on cancer risk was found with maternal proteinuria, which may be too crude a measure of pre-eclampsia, maternal blood pressure at delivery not being recorded. On the other hand, maternal proteinuria was highly negatively associated with weight at birth (data not shown) in the current study. Low weight at birth, a possible consequence of maternal pre-eclampsia, may be the determining factor in the relation rather than the pre-eclampsia per se.

Age at menarche may be an explanatory factor for hormone-related cancer risk and size at birth but was available for only a portion of our sample; it cannot be excluded as a possible important covariate (Hsieh et al, 1990).

If size at birth is a risk factor for later disease, what factors predispose for large size? Larger size at birth may be a consequence of hyperinsulinism due to impaired maternal glucose tolerance or gestational diabetes (D’Ercole, 1999). In this study maternal glucose measurements were not routinely made during the study period. In our own review of hospital births for this period (1914–1930), no mother was recorded as diabetic before or during the period of pregnancy and women with diabetes were most probably advised against pregnancy at that time period, if they indeed survived into adulthood with childhood diabetes. Large size at birth generally reflects genetic propensity based on maternal and paternal genotype rather than pathological overgrowth (D’Ercole, 1999). It cannot be excluded that an as yet unknown growth factor-X may lead to increased fetal growth and/or increased risk for cancer. Insulin-like growth factor II (IGF-II) plays a fundamental role in human fetal growth and has been implicated to be involved in human tumorigenesis (O’Dell and Day, 1998). Growth factors at birth were not assessed in our study.

Lack of statistical significance may be attributed to low power due to the small number of cases when looking at individual or limited groupings of cancer sites in this study. Despite lack of significance in some of the modelling, there are strong suggestions of an increase in cancer risk with higher birth weight and birth length.

At the population study onset, the participation rate was 90.6% (Bengtsson et al, 1973), and all women identified in the

*© 2001 Cancer Research Campaign* 

complementary population could be followed from birth to endpoint by tracing parish records throughout the lifespan. Original birth records could be traced for 68% of the women in the study population. Access to original midwife records and parish records has the advantage of eliminating recall bias and misclassification since we do not rely upon self-reported birth data (Andersson et al, 2000a). An earlier study in the same population material found the Swedish Cancer Registry database to have captured 99% of cancer cases (Helgesson et al, 1994) attesting to completeness of the cancer endpoint. Aside from age at menarche which was attained by questionnaire, all variables were extracted from original records.

The present findings can be seen from 2 viewpoints: (a) there is a higher risk for cancer with higher birth weight or, (b) there is a protective effect of low birth weight on cancer risk independent of gestational age. In either case, the balance of evidence suggests that size at birth may require consideration in the pathogenesis of adult cancers. Our findings warrant further study in larger data sets.

### ACKNOWLEDGEMENT

Sources: The Swedish Medical Research Council (B96-27X-11659 and B96-19X-07509), The Swedish Society of Medicine, Faculty of Medicine Göteborg University, Sahlgrenska University Hospital Research Fund and the Wilhelm and Martina Lundgren Foundation.

### REFERENCES

Andersson SW, Niklasson A, Lapidus L, Hallberg L, Bengtsson C and Hulthén L (2000a) Poor agreement between self-reported birth weight and birth weight from original records in adult women. *Am J Epidemiol* **152**:

Andersson SW, Niklasson A, Lapidus L, Hallberg L, Bengtsson C and Hulthén L (2000b) Sociodemographic characteristics influencing birth outcome in Sweden, 1908–1930. Birth variables in the Population Study of Women in Gothenburg. *J Epidemiol Community Health* **54**: 269–278

Bengtsson C, Blohme G, Hallberg L, Hallstrom T, Isaksson B, Korsan-Bengtsen K, Rybo G, Tibblin E, Tibblin G and Westerberg H (1973) The study of women in Gothenburg 1968–1969 – a population study. General design, purpose and sampling results. *Acta Med Scand* **193**: 311–318

Brown LM, Pottern LM and Hoover RN (1986) Prenatal and perinatal risk factors for testicular cancer. *Cancer Res* **46**: 4812–4816

Daling JR, Starzyk P, Olshan AF and Weiss NS (1984) Birth weight and the incidence of childhood cancer. *J Natl Cancer Inst* **72**: 1039–1041

D’Ercole AJ (1999). Mechanisms of in utero overgrowth. *Acta Paediatr Suppl* **88**: 31–36

Ekbom A (1998). Growing evidence that several human cancers may originate in utero. *Semin Cancer Biol* **8**: 237–244

Ekbom A, Trichopoulos D, Adami HO, Hsieh CC and Lan SJ (1992) Evidence of prenatal influences on breast cancer risk [see comments]. *Lancet* **340**: 1015–1018

Ekbom A, Hsieh CC, Lipworth L, Wolk A, Ponten J, Adami HO and Trichopoulos D (1996) Perinatal characteristics in relation to incidence of and mortality from prostate cancer. *BMJ*, **313**, 337–341

Ekbom A, Hsieh CC, Lipworth L, Adami HQ and Trichopoulos D (1997) Intrauterine environment and breast cancer risk in women: a population-based study *J Natl Cancer Inst*, **89**: 71–76

Helgesson O, Bengtsson C, Lapidus L, Merck C and Sparen P (1994) Malignant disease observed in a cohort of women. A validation of Swedish Cancer Registry data. *Scand J Soc Med* **22**: 46–49

Hsieh CC, Trichopoulos D, Katsouyanni K and Yuasa S (1990) Age at menarche, age at menopause, height and obesity as risk factors for breast cancer: associations and interactions in an international case-control study [see comments]. *Int J Cancer* **46**: 796–800

Janerich DT, Hayden CL, Thompson WD, Selenskas SL and Mettlin C (1989) Epidemiologic evidence of perinatal influence in the etiology of adult cancers. *J Clin Epidemiol* **42**: 151–157

Joseph KS and Kramer MS (1996) Review of the evidence on fetal and early childhood antecedents of adult chronic disease. *Epidemiol Rev* **18**: 158–174

Lynch HT, Fusaro RM and Lynch JF (1997) Cancer genetics in the new era of molecular biology. *Ann N Y Acad Sci* **833**: 1–28

Michels KB, Trichopoulos D, Robins JM, Rosner BA, Manson JE, Hunter DJ, Colditz GA, Hankinson SE, Speizer FE and Willett WC (1996) Birthweight as a risk factor for breast cancer [see comments]. *Lancet* **348**: 1542–1546

Miller AB (1978) An overview of hormone-associated cancers. *Cancer Res* **38**: 3985–3990

O’Dell SD and Day IN (1998) Insulin-like growth factor II (IGF-II). *Int J Biochem Cell Biol* **30**: 767–771

Potischman N and Troisi R (1999) In-utero and early life exposures in relation to risk of breast cancer. *Cancer Causes Control* **10**: 561–573

Potter JD (1995) Hormones and colon cancer [editorial; comment]. *J Natl Cancer Inst* **87**: 1039–1040

Sanderson M, Williams MA, Malone KE, Stanford JL, Emanuel I, White E and Daling JR (1996) Perinatal factors and risk of breast cancer. *Epidemiology* **7**: 34–37

Socialstyrelsen NBoHaW (1997) *Cancer incidence in Sweden 1994*. Official Statistics Sweden: Stockholm.

Stavola B, Hardy R, Kuh D, dos Santos Silva I and Swerdlow A (2000) Birthweight, childhood growth and risk of breast cancer in a British cohort. *Br J Cancer* **83**: 964–968

Tibblin G, Eriksson M, Cnattingius S and Ekbom A (1995) High birthweight as a predictor of prostate cancer risk [see comments]. *Epidemiology* **6**: 423–424

Trichopoulos D (1990) Hypothesis: does breast cancer originate in utero? [see comments]. *Lancet* **335**: 939–940

Yeazel MW, Ross JA, Buckley JD, Woods WG, Ruccione K and Robison LL (1997) High birth weight and risk of specific childhood cancers: a report from the Children’s Cancer Group. *J Pediatr* **131**: 671–677

 *© 2001 Cancer Research Campaign*
